# Cariprazine for Clozapine-Induced Urinary Incontinence in Treatment-Resistant Depression: A Case Report

**DOI:** 10.7759/cureus.77582

**Published:** 2025-01-17

**Authors:** Ana Carolina Pires, Isabela Faria, Joana Marques Pinto, Miguel Bajouco, Joana Andrade

**Affiliations:** 1 Psychiatry, Unidade Local de Saúde de Coimbra, Coimbra, PRT

**Keywords:** antipsychotic agents, cariprazine, clozapine, mood disorders, treatment-resistant depressive disorder, urinary incontinence

## Abstract

Urinary incontinence (UI) is an adverse effect associated with clozapine treatment. Several management options are available, including adjuvant medication. We describe the case of a woman in her 50s with a history of major depressive disorder and a current severe depressive episode with psychotic symptoms. The case is discussed, and a review of the relevant literature is presented, examining the use of cariprazine in the management of clozapine-induced UI. Cariprazine was initiated as a first-line treatment, followed by clozapine several months later. Following a reduction in the dose of cariprazine, the patient developed UI. Physical examination was unremarkable, and laboratory investigations were normal. No additional interventions were required. No other medication was altered, and upon reintroduction of cariprazine, the patient's UI improved significantly. Although a single case is inadequate for establishing definitive conclusions, this case report indicates the need for further investigation into the potential of cariprazine in addressing clozapine-induced UI.

## Introduction

Clozapine is an effective atypical antipsychotic used in the management of treatment-resistant schizophrenia and the prevention of suicidal behaviors in patients diagnosed with schizophrenia or schizoaffective disorder. Additionally, it has been used off-label in patients with other disorders, such as treatment-resistant major depressive disorder (MDD). It has been shown that clozapine is associated with a reduced risk of hospital readmission in patients with MDD and may be more effective than other antipsychotics in treating depressive symptoms in these patients [1].

Clozapine has a complex mechanism of action and acts on several receptors. This molecule acts on the dopaminergic receptors D1, D2, D3, and D4, with a higher affinity for D4. Clozapine also binds to the adrenergic receptors α1 (A and B) and α2 (A, B, and C), showing a higher affinity for the α1. Concerning serotonin (5-hydroxytryptamine) receptors, it has an action on 5-HT1A, 5-HT1B, 5-HT1D, 5-HT1E, 5-HT2A, 5-HT2B, 5-HT2C, 5-HT3, 5-HT6, and 5-HT7, with a stronger binding profile to 5-HT2B and 5-HT2A. Clozapine also binds to muscarinic receptors M1, M2, M3, and M4 [2]. Clozapine has shown antidepressant-like effects in a rat model, improving despair and anhedonia, restoring neuronal function in the hippocampus and prefrontal cortex, and promoting neurogenesis. It has also been shown to contribute to increased BDNF levels in the frontal cortex, like antidepressants, contributing to its therapeutic potential [1].

Urinary incontinence (UI) is a potential adverse drug reaction (ADR) of clozapine treatment. The prevalence of clozapine-induced nocturnal enuresis can be as high as 10%-42%. Furthermore, clozapine is among the antipsychotic agents with the highest likelihood of inducing UI [3]. While some patients experience spontaneous resolution, others require intervention to mitigate the impact on the quality of life and avoid treatment discontinuation [4]. 

The mechanisms by which clozapine can trigger UI are not completely understood. The high affinity of clozapine to antagonize α1 receptors may contribute to internal urethral sphincter relaxation [2,5,6]. Clozapine also exhibits anticholinergic properties [2], which can cause adverse effects, such as constipation or, in more severe cases, paralytic ileus. However, clozapine does not appear to be associated with the expected incidence of urinary retention. This may be because its main metabolite (N-desmethylclozapine) is a muscarinic receptor partial agonist [5]. However, some authors consider that the anticholinergic effects of clozapine can induce partial urinary retention, resulting in overflow incontinence [7].

Another proposed mechanism involves the partial agonism of clozapine on 5-HT1A receptors [8]. The prefrontal cortex (PFC) is involved in complex mechanisms of inhibitory control of micturition and tonic suppression of voiding. Some authors have argued that activating 5-HT1A receptors can increase PFC activity and lead to increased action of the detrusor muscle. The detrusor muscle of the bladder remains relaxed to allow it to accumulate urine and contracts to eliminate urine [9]. Activation of 5-HT1A receptors has been shown to lower the micturition reflex threshold and prolong urethral sphincter relaxation [10]. Some authors argue that this receptor can have different actions in different species and that its role is not yet fully understood [11]. The antagonistic action on 5-HT2 and 5-HT3 receptors can lead to the blockage of the pudendal reflexes, resulting in UI [6,7]. In addition to these mechanisms, the effects on dopamine transmission in the basal ganglia may also play a key role in clozapine-induced UI [6].

Clozapine-induced UI presents a challenge in terms of therapeutic management, with no official guidelines currently in place. First-line options include non-pharmacological measures, such as the use of urinary alarms, frequent urination, urination before bedtime, and fluid restriction. Pharmacologic management options are second-line and include switching to other antipsychotics with fewer anticholinergic effects, a reduction in the dose of clozapine, and the use of adjuvant medications. Several drugs have been identified as potential treatments, such as desmopressin, oxybutynin, imipramine, amitriptyline, verapamil, and second-generation antipsychotics [3,4,7].

To date, no studies have investigated the use of cariprazine for the management of clozapine-induced UI. Regarding the potential effect of cariprazine on urinary function, to our knowledge, only one case of urinary retention has been described in the current literature [12]. In this context, aripiprazole, a molecule with similar pharmacological properties, has been explored. Case reports suggest that aripiprazole, at doses of 10-15 mg/day in combination with clozapine, may reduce UI [4,6,13,14].

Cariprazine is a third-generation antipsychotic approved for schizophrenia, bipolar type I disorder, and major depressive disorder. It is a partial agonist for D3 and D2 receptors, with a higher affinity for D3 [15]. It also binds to 5-HT1A, 5-HT2A, 5-HT2B, 5-HT2C, and 5-HT7 receptors. It acts on adrenergic α1 (A, B, and D) and α2 (A) receptors as well as muscarinic M1 receptors [2]. The similar receptor profiles of cariprazine and aripiprazole suggest they may share mechanisms for managing clozapine-induced UI, particularly through their partial agonist activity at D2 and D3 receptors and modulation of serotonin pathways. However, cariprazine's distinct receptor affinity may offer unique therapeutic advantages.

This case report presents a woman in her 50s who exhibited a positive response to cariprazine in the management of clozapine-induced UI. A review of the existing literature on the topic is conducted to contextualize this clinical case, aiming to encourage further research in this area.

## Case presentation

The patient was a woman who was diagnosed with MDD. She held a graduate degree and worked in a full-time skilled position. She lived in an urban area with her nuclear family (her husband and her only child who was 23 years old). The current episode began when she was 51 years old. Two months after the onset of the complaints, she had a psychiatric appointment, and venlafaxine was started, titrating it to 225 mg/day, as well as trazodone 150 mg/day, with adequate therapeutic compliance. Two months later, she was admitted to the psychiatric emergency department following a suicide attempt by hanging. The patient presented with depressed mood, anhedonia, hypersomnia, psychomotor agitation, anergia, poor concentration, and active suicidal ideation. The patient was subsequently admitted to the psychiatric ward. At admission, the antidepressant treatment was adjusted, and a switch to fluoxetine was made, titrated to 60 mg/day, and quetiapine 200 mg/day was initiated. Psychomotor agitation and hypersomnia were controlled, but the remaining symptoms persisted. Two months after admission, given the severity of her clinical presentation, adjunctive treatment was initiated with bupropion 150 mg/day and lithium up to 800 mg/day. There was a lack of a satisfying clinical response after two trials of antidepressant medication of different classes at therapeutic doses (fluoxetine and venlafaxine) for a minimum of four weeks each, as well as with the adjunctive medication. Electroconvulsive therapy (ECT) was offered to the patient. Beginning in the eighth month of the current episode, a total of 17 sessions of ECT were completed, but an adequate seizure was not obtained in five of them. Although there was a partial clinical response, ECT was ceased due to the onset of severe bradycardia. Quetiapine was stopped. Five months after admission, the patient was transferred to a psychiatric day hospital with fluoxetine 60 mg/day, bupropion 150 mg/day, lithium 800 mg/day, and trazodone 150 mg/day.

Upon admission to the day hospital, the patient presented with secondary delusions of guilt, and cariprazine (3 mg/day) was initiated. In the 11^th^ month of the current episode, as the symptoms partially persisted, clozapine was initiated, which was titrated up to 125 mg/day. The patient responded to clozapine concerning psychotic and depressive symptoms. One month after this therapy, cariprazine was progressively discontinued to avoid polypharmacy, given the partial improvement of her symptoms.

Two weeks after gradually discontinuing cariprazine, the patient complained of daily UI, sometimes several times a day. These episodes were classified as urgency UI subtype. The patient described them as having an unexpected and intense need to urinate, resulting in involuntary urine loss of variable volume. It occurred both during the day and at night, some of which were witnessed by the medical and nursing staff during her stay in the day hospital. They were not associated with any urinary symptoms or physical exertion. She had never experienced the involuntary passing of urine previously. No other medication was changed during this period.

The patient had a personal history of MDD. Her first depressive episode was severe and postpartum, occurring at the age of 28 years old. During this episode, the patient attempted suicide by jumping from a high altitude, resulting in splenic trauma and subsequent admission to the general surgical unit. Once her condition had stabilized, she was transferred to the psychiatric ward for two months. After her discharge, the patient was followed up with psychiatric appointments. She was prescribed fluoxetine 40 mg/day during her hospitalization, which was later reduced to 20 mg/day and continued for a decade. At 43 years of age, the patient experienced a new depressive episode of moderate severity, which was managed with ambulatory care. The dose of fluoxetine was increased to 40 mg/day, and a year later it was reduced again to 20 mg/day. She stopped taking it one year before the current episode. No records indicated the presence of manic or hypomanic episodes throughout the patient's lifespan.

There was no history of prior iatrogenic drug effects. Her medical history revealed that she had controlled diabetes and hypertension, taking the same medication for years: metformin 850 mg/day and amlodipine 5 mg/day. Aside from her gender, age, and excessive weight, the patient had no other risk factors for urinary incontinence. No other significant medical or surgical history. She was a non-smoker and had no other addictive behaviors. There was no evidence of a family history of psychiatric illness or UI.

Electroencephalography and cranio-encephalic magnetic resonance imaging did not reveal any alterations. Blood and urine analyses showed no abnormalities, including glycated hemoglobin, fasting blood glucose levels, and lithium blood levels. Physical examination revealed no abnormalities. As urinary tract infection was ruled out, the case was discussed with the urology and gynecology specialists, suggesting no further intervention other than managing clozapine-induced UI.

The episodes of UI could not be adequately explained by other known factors, including the patient’s current clinical condition, medical history, or other medications. Cariprazine was reintroduced, initially at a dose of 1.5 mg/day and later increased to 3 mg/day. This led to a gradual and sustained reduction in the frequency of UI episodes, which decreased to a maximum of two episodes per month. Bupropion and trazodone were later discontinued. The patient was subsequently discharged from the day hospital in the 13^th^ month of the episode and continued to attend psychiatric appointments for another six months. At the time of discharge, she was prescribed fluoxetine 60 mg/day, lithium 800 mg/day, cariprazine 3 mg/day, and clozapine 50 mg/day. The number of UI episodes remained minimal during this period. Clozapine was stopped during the ambulatory follow-up appointments, and the UI episodes ceased. She returned to her job in the 17^th^ month of the depressive episode.

## Discussion

This case study concerns a patient with MDD, who experienced a treatment-resistant severe episode with psychotic symptoms. Multiple medication adjustments were required during the admission to the psychiatric ward and, later, to the day hospital.

According to the World Health Organization-Uppsala Monitoring Centre (WHO-UMC) system for causality assessment [16], it is certain in this case that the UI was an ADR of clozapine, which was effectively managed with cariprazine. The patient did not report any complaints of UI before admission or after, while taking the prescribed medications, including fluoxetine, bupropion, lithium, and trazodone, nor when cariprazine was introduced. Following the initiation of clozapine treatment, no episodes of urine loss were observed. UI episodes began only after the discontinuation of cariprazine, while clozapine treatment was ongoing. There is a temporal association between the discontinuation of cariprazine and the onset of UI, as well as between the reintroduction of cariprazine and a significant reduction in UI episodes. The most likely explanation for the UI is the ADR of clozapine, given the absence of urinary tract infection, structural abnormalities in the central nervous system, or other contributing factors, such as uncontrolled diabetes. The patient’s case was also reviewed by gynecology and urology specialists, who found no alternative explanations for her symptoms and discharged her. Clozapine-induced UI is well-documented in the literature [3,4].

Regarding the ADR Probability Scale by Naranjo [16], the total score was 11, indicating that the UI episodes were a definite reaction to clozapine. These episodes were witnessed by healthcare professionals and exhibited a clear temporal relationship with the initiation of clozapine and discontinuation of cariprazine. This reaction, recognized as a clozapine-induced ADR, was further substantiated by the reintroduction of cariprazine and subsequent withdrawal of clozapine.

Clozapine-induced UI mechanisms are complex, and there are no official guidelines for its management. Second-generation antipsychotics are an option, and case reports suggest that aripiprazole may reduce clozapine-induced UI [4,6,13,14]. Enuresis is associated with hypodopaminergic and hypoadrenergic states. Aripiprazole is a D2 receptor partial agonist, so it acts as an agonist in the hypodopaminergic basal ganglia during the clozapine treatment. An increase in the activation of D2/D3 receptors can potentially reduce the UI. Literature suggests that dopamine also has the potential to cross-activate adrenoceptors, both central and peripheral, such as α1 adrenoreceptors, probably resulting in improved sphincter tone [17]. Compared to clozapine, aripiprazole has a reduced affinity for α1-adrenergic receptors, resulting in a diminished antagonistic effect [4,6,18]. Aripiprazole, like clozapine, is a 5-HT1A partial agonist. However, they have very different affinities for this receptor, with aripiprazole being more potent than clozapine [19]. Thus, some authors have suggested that aripiprazole could reduce bladder dysfunction compared with clozapine [12,13]. In addition, aripiprazole has a negligible antimuscarinic effect, making it less likely to induce partial urinary retention [18], which could result in overflow incontinence [6]. Although aripiprazole also has an antagonistic action on 5-HT2 receptors, even with greater affinity than that of clozapine [18], the overall effect of this pharmacological molecule appears to improve UI. Furthermore, some authors claim that the antagonism of 5-HT2 receptors could inhibit bladder contraction [12], potentially counteracting UI, despite its impact on pudendal reflexes [6,7].

There is a case report of urinary retention associated with the use of cariprazine [12], which may represent its counterbalance to clozapine-induced UI. Cariprazine is a very similar molecule to aripiprazole, and both are considered third-generation antipsychotics because of their partial agonistic actions on the dopamine receptors. The effects of cariprazine treatment on clozapine-induced UI may be attributed to its action as a partial D2/D3 agonist, given its capacity to regulate dopaminergic activity in the basal ganglia, thereby controlling UI [17]. The partial agonism of cariprazine on the 5-HT1A receptors is very similar to that of aripiprazole [20], which may contribute to the reduction of bladder dysfunction [6,13]. Cariprazine is thought to have negligible affinity for muscarinic and adrenergic receptors [12], like aripiprazole. This reduces the likelihood of overflow incontinence [6]. Additionally, cariprazine has been demonstrated to exert antagonistic effects on 5-HT2 receptors, which may inhibit bladder contraction [12] and thereby prevent UI. While these mechanisms have been implicated in UI mitigation, additional unexplored pathways might also contribute.

The similarity in receptor profiles between cariprazine and aripiprazole suggests that they may have similar mechanisms for addressing UI. However, cariprazine's specific affinity profile for receptors may provide unique benefits. This case represents the first report of cariprazine's role in this domain, underscoring its potential as a therapeutic strategy for clozapine-induced UI when non-pharmacological approaches prove ineffective. Introducing cariprazine early in the treatment of clozapine-induced UI, particularly for patients with treatment-resistant MDD, could be a viable option. The balance between benefits and risks must be carefully evaluated, given the need to minimize polypharmacy.

The main strengths of this case report are the thorough clinical observations and the innovative application of cariprazine, which is supported by the current literature. However, several limitations must be acknowledged. As a single case report, the findings cannot establish causation or generalizability. Additionally, the absence of objective measures for UI severity limits the precision of symptom quantification. Future investigations should include controlled studies with larger sample sizes and standardized metrics for UI assessment to substantiate these findings.

## Conclusions

This case report underscores the potential of cariprazine as a therapeutic option for treating clozapine-induced UI. Based on established ADR causality assessment methods, there is a definite association between UI and clozapine in this case. There appears to be a temporal link between the discontinuation of cariprazine and the onset of UI, as well as between its reintroduction and a significant reduction in UI episodes. Given cariprazine's similarities to aripiprazole and the evidence supporting the latter's efficacy in similar contexts, cariprazine may offer a promising therapeutic option. Further research is required to ascertain its efficacy in the management of clozapine-induced UI to develop tailored pharmacological strategies and improve patient outcomes.
